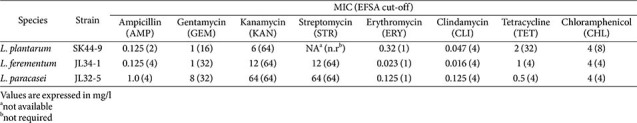# Development of a Novel Probiotic Strain with Digestion Functionality, *Lacticaseibacillus paracasei* JL32-5 from Chorizo

**DOI:** 10.4014/jmb.2512.12007

**Published:** 2025-12-29

**Authors:** Li-Ha Kim, Suhyeon Park, Jae-Hee Kwon, Ju-Hoon Lee

**Affiliations:** 1 Department of Agricultural Biotechnology, Seoul National University, Seoul 08826, Republic of Korea; 2 Department of Food and Animal Biotechnology, Seoul National University, Seoul 08826, Republic of Korea; 3 Center for Food and Bioconvergence, Seoul National University, Seoul 08826, Republic of Korea; 4 Research Institute of Agriculture and Life Sciences, Seoul National University, Seoul 08826, Republic of Korea

**Keywords:** Probiotics, lipase activity, proteolytic activity, xylan utilization, probiotic safety, probiotic stability

## Abstract

Several *Lactobacillus* strains isolated from fermented food are expected to possess digestion supporting function as potential probiotics. However, the safety of *Lactobacillus* as functional food and its stability until consumption are often overlooked. To prevent potential side effects and maximize probiotic efficacy, it is essential not only to demonstrate the beneficial properties of candidate strain but also verify their safety and stability through a stepwise method. In this study, *Lactobacillus* strains were isolated from various fermented food and sequentially evaluated for safety, stability, and probiotic properties with a focus on digestive activity. First, five safety tests were conducted in accordance with WHO/FAO guidelines-antibiotic minimum inhibitory concentrations (MICs), hemolytic activity, bile salt hydrolase (BSH) activity, D-/L-lactate production, and cytotoxicity based on lactate dehydrogenase (LDH) released from human epithelial cells. Next, four stability tests were performed, including tolerance to heat, oxygen, and gastric acid/bile salts. In addition, adhesion ability to intestinal epithelial cells was examined as an indicator of colonization potential. Finally, probiotic properties related to assistance of digestion–lipase activity, protease activity, lactose and xylan utilization ability—were evaluated. Through this three-step evaluation, *Lacticaseibacillus paracasei* JL32-5 was proven to be a safe and stable probiotic resource supporting digestion of lipid, protein, lactose, and non-digestible carbohydrate therefore enhancing human wellness.

## Introduction

Modern dietary patterns, excessive intake of refined nutrients combined with chronically low fiber consumption, have led to an increase in digestive disorders [1]. In fact, diseases associated with maldigestion such as pancreatic insufficiency and lactose intolerance are commonly reported. The global prevalence of exocrine pancreatic insufficiency (EPI), a condition of impaired macronutrient breakdown caused by low pancreatic enzyme secretion [2], is estimated around 20% [3]. Pancreatic secretions are composed of proteases (80%), amylase (7%), and lipase (4%), therefore EPI patients undergo maldigestion [4]. Lactose intolerance, insufficient activity of lactase leads to lactose undigested, is frequently reported from over 65% of adults [5]. Also, unlike healthy people, in those who suffer from short bowel syndrome, carbohydrates are not fully digested [6] in small intestine. As a result of maldigestion, partially digested or intact macronutrients reach the large intestine and serve as substrates for intestinal bacteria. Upon fermentation, gaseous metabolites including carbon dioxide and methane are produced and contribute to gastrointestinal discomfort such as bloating, abdominal pain, or dyspepsia [7]. In severe cases, the condition may progress to diseases such as steatorrhea and biotin deficiency. Steatorrhea, defined as an abnormal increase in fecal fat content over 7 g per day, accompanies pale, bulky, malodorous, and loose stools [8]. Also, undigested avidin, an abundant protein of egg white, can sequester biotin, leading to biotin deficiency and changes in gut microbiota composition followed by irritable bowel disease (IBD)-like phenotype [9]. In addition, wheat bran containing arabinoxylan hemicellulose as the major non-starch polysaccharide [10] serves as a beneficial dietary fiber in healthy individuals, but has been reported to exacerbate discomfort and might be not suitable for the IBD patients [11].

Maldigestion syndromes have been managed with dietary modification and enzyme supplementation of lipase, protease, or lactase. For instance, susceptible people to lactose limit dairy intake and consume lactose-free products instead. Also, EPI patients consume low-fat and low-fiber diet [12]. Dietary modification can prevent uncomfortable gastrointestinal symptoms or potential health issues. However, this approach is a temporary avoidance and is not sustainable for long-term application, due to the risk of malnutrition. In fact, the pancreatic enzyme replacement therapy (PERT), the standard treat for EPI, also has limitations, with residual nutritional deficiencies reported in nearly two-thirds of patients [4]. Moreover, excessive use of digestive enzyme supplements can increase dependency and potentially disrupt normal digestive processes [13], and is associated with side effects such as nausea, abdominal cramping, and gastritis [14]. Therefore, there has been a growing demand of functional food as daily supplements that modulate intestinal digestion to alleviate nondigestive disorders related to complex carbohydrate, protein, lipid, and lactose metabolism without potential undesirable effects.

With the discovery of the central role of gut microbiota, probiotics defined as “live microorganisms which, when administered in adequate amounts, confer a health benefit on the host” (FAO/WHO) have emerged as a novel approach to address maldigestion in macronutrient metabolism. In addition, advancements of microbiome research in characterization of strain-specific and origin-dependent functionalities [15] have enabled the development of more efficient probiotics against chronic diseases [16]. In fact, several clinical studies have administered probiotics to patients and evaluated their ability to alleviate gastrointestinal symptoms, demonstrating symptom-relieving effects. For instance, *Bifidobacterium longum* subsp. *longum* and *Limosilactobacillus reuteri* supplementation can improve intestinal conditions toward the amelioration of lactose intolerance [17]. Also, the effects of probiotics have been evaluated on patients with predicted severe acute pancreatitis (SAP) [18]. However, current evaluation methods rely on clinical studies assessing symptom relief, and the available data remain insufficient to draw definitive conclusions about probiotic effects, leaving the functional efficacy unclear. Therefore, new strategies to elucidate the probiotic function degrading specific non-digestible macronutrients must be applied.

In general, *Lactobacillus* is one of the most used genera and 11 species (*Lactobacillus acidophilus*, *Lacticaseibacillus casei*, *Lactobacillus delbrueckii* subsp. *bulgaricus*, *Limosilactobacillus fermentum*, *Lactobacillus gasseri*, *Lactobacillus helveticus*, *Lacticaseibacillus paracasei*, *Lactiplantibacillus plantarum*, *Limosilactobacillus reuteri*, *Lacticaseibacillus rhamnosus*, *Ligilactobacillus salivarius*) among 260 *Lactobacillus* species [19] were officially classified as probiotics by the Korean Ministry of Food and Drug Safety (MFDS). Because of long history of safe consumption [20] as functional food and prevalence in both human gut microbiota and traditional fermented food [21], *Lactobacillus* is regarded as an attractive genus for probiotics development. Fermented food containing high levels of fat, protein, complex polysaccharides or lactose substantially differ from the environment of human gastrointestinal tract. Therefore, *Lactobacillus* derived from fermented food is expected to possess distinct metabolic and physiological traits [22] such as protease, lipase, lactase, or polysaccharide degrading enzyme activities. Meanwhile, the increasing consumption of probiotic *Lactobacillus* strains by individuals with diverse health conditions-including immunocompromised or otherwise vulnerable populations has raised concerns about potential adverse effects [23], even though *Lactobacillus* has long been regarded as a Generally Recognized as Safe (GRAS). Therefore, comprehensive safety validation of probiotic candidates must be performed following WHO/FAO, EFSA, OECD, and MFDS guidelines (2021) as a prerequisite before clinical or commercial application. Alongside probiotic safety, physiological robustness, especially tolerance in temperature and oxygen, of probiotics directly influence production efficiency [24]. In fact, application of optimal culture temperature is essential for high survival of probiotics during mass production [25] and usage of oxygen tolerant probiotic strains ensures high cell counts in products during storage [26]. Furthermore, for probiotics to exert functional activities in the host, it must withstand multiple harsh conditions of gastrointestinal tract gastric acid secreted from stomach and bile salts in small intestine [27]-and finally adhere to host epithelial cells [28], interacting to host and another gut microbiota through various mechanisms. Considering this, culturing probiotic candidates in stressful conditions reflecting industrial processing, storage, and human gastrointestinal tract might be applied to select the most stable candidate. For the digestion supportive functional analysis, lipid, protein, lactose, and xylan can serve as general substrates. Through evaluation of abilities that degrade or utilize various macronutrients, strain-specific digestive supporting ability can be discovered.

In this study, digestive-supporting probiotics were systematically developed through four sequential steps: isolation from fermented food, safety test, stability analysis, and functional evaluation related to digestive support. At first, *Lactobacillus* from fermented food was selected as potential probiotic candidates. Next, three isolates were subjected to safety validation. After that, probiotic stability tests were performed. Finally, digestive supportive abilities were evaluated against diverse macronutrients. Lipase activity was evaluated using triglycerides as substrate. Commonly consumed protein types, milk protein concentrate (MPO), whey protein concentrate (WPO), casein, and soy, were used as substrates of bacterial proteolytic activity. Xylan, one of a major hemicellulose derived from cereals, fruits, and vegetables and particularly challenging to digest, due to lack of xylanase activity was used for the bacterial digestion ability against complex carbohydrates. Consequently, this approach enables the development of functionally explicit probiotic candidates that, when consumed, can help prevent symptoms associated with poor digestibility.

## Materials and Methods

### *Lactobacillus* Isolation from Fermented food

Fifty gram of food sample and 100 ml of 0.1% (w/v) sterilized peptone (Difco, USA) water were loaded in a stomacher bag and mixed thoroughly using a stomacher (BNF, Republic of Korea). The homogenized sample was inoculated into MRS-c broth (Difco) supplemented with 0.05% (w/v) L-cysteine hydrochloride (Sigma-Aldrich, USA), at 1% (v/v) concentration for *Lactobacillus* enrichment. After 48 h incubation, incubates were serially diluted with Dulbecco’s phosphate buffer (GenDEPOT, USA) at 10-fold ratio and inoculated onto LBS (MBCell, Republic of Korea) agar plates. After incubation, representative colonies were picked for further identification.

### *Lactobacillus* Identification

Three isolates were identified in species-level based on 16S rRNA region. Colony PCR was performed using Taq polymerase (TaKaRa, Japan) following manufacturer’s instruction. The 16S rRNA gene was amplified by PCR using 27F (5’-AGA GTT TGA TCM TGG CTC AG-3’) and 1492R (5’-CGG TTA CCT TGT TAC GAC TT-3’) primers as follows; Pre-denaturation at 98°C for 3 min, denaturation at 98°C for 10 sec, annealing at 55°C for 1 min, extension at 72°C for 1 min (30 cycles), and final-extension at 72°C for 3 min. The PCR products were mixed with 1.5 X loading dye (Bionics, Republic of Korea) and loaded in a 0.8% (w/v) agarose gel. 1 kb DNA ladder (Bio-Rad, USA) was used as a reference of amplicon size. Amplicons were visualized by GelDoc (Bio-Rad) under UV light to validate 1.5 kb length of the PCR product. After that, PCR products were purified with PCR product purification kit (Bionics) following the manufacturer’s instruction. The nucleotide sequences in 16S rRNA region were read by sanger sequencing (Bionics) using 1492R primer. After that, the identity was confirmed by manual alignment of quality assured sequence to sequence database of National Center for Biotechnology Information (NCBI) using Basic Local Alignment Search Tool–nucleotide (BLASTN) [29]. All identified isolates were stored in a deep freezer as a frozen stock made of MRS-c broth and 80% (v/v) glycerol for further usage.

### Bacterial Culture

Culture media was sterilized at 121°C for 15 min before usage and agar media was supplemented with 1.8% (w/v) bacto agar (Difco) before sterilization if not mentioned. Probiotic candidates were revived from a cryovial by streaking on MRS-c agar for *Lactobacillus* and Tryptic Soy Agar (TSA; Difco) for non-*Lactobacillus* species, respectively, to obtain single colonies. After incubation, representative colony was inoculated into broth media. *Lactobacillus* strains were cultured under anaerobic and static conditions using anaerogen (Oxoid, USA) and anaerobic jar (BD, USA) at 37°C unless otherwise noted. For cultivation of non-*Lactobacillus* species, aerobic and static culture at 37°C was used.

### Probiotic Safety Evaluation

**Antibiotic susceptibility test.** The lactic acid bacteria (LAB) susceptibility test medium (LSM; composed of 90% (v/v) Iso-sensitive broth (MBCell) and 10% (v/v) MRS broth (Difco) supplemented with 0.05% (w/v) L-cysteine hydrochloride (Sigma-Aldrich) and 0.6% (w/v) Bacto agar (Difco)) was used for MIC test of probiotic candidates [30]. Bacterial culture was inoculated to LSM at 1% (v/v) ratio and poured into petri dish (SPL, Republic of Korea). E-strips (bioMérieux, France) containing gradient concentration of antibiotics were placed on solidified agar. Probiotic candidates were tested against required antibiotics. Resistance against eight antibiotics including ampicillin (AMP), gentamicin (GEM), kanamycin (KAN), streptomycin (STR), erythromycin (ERY), clindamycin (CLI), tetracycline (TET), and chloramphenicol (CHL) was evaluated for *L. paracasei* and *L. rhamnosus*. Seven antibiotics excluding streptomycin (STR) were tested for *L. plantarum*. Inoculates were incubated as mentioned before. The lowest concentration that no bacterial growth on each strip was determined as the MIC. When MIC was within EFSA’s cut-off criteria, candidates were regarded as sensitive to antibiotics phenotypically.

### Hemolysis Assay

Probiotic candidates were streaked onto TSA (Difco) supplemented with 5% (v/v) sheep blood defibrinated (MBCell) followed by incubation for 24 h. *Streptococcus salivarius* ATCC 19250, *Staphylococcus aureus* ATCC 29213, and *Staphylococcus epidermis* ATCC 35983 were used as positive control for *α-*, *β-*, and *γ-*hemolysis, respectively. The hemolytic phenotypes of all tested strains were observed under light plate.

### Bile Salt Hydrolase (BSH) Activity Assay

BSH agar composed of MRS-c supplemented with 0.5% (W/V) Taurodeoxycholic acid (TDCA; Sigma-Aldrich) was prepared. Probiotic candidates were streaked onto BSH agar and incubated for 24 h. *L. plantarum* 182 and *L. rhamnosus* GG (ATCC 53103) were used as positive and negative strain, respectively.

### Lactic Acid Production Profiling

Secreted lactic acid in cell free supernatant (CFS) of *Lactobacillus* culture media after 24 h incubation, a stationary point at which metabolites were abundantly produced, was measured based on colorimetric assay. CFS was prepared by filtering homogenized culture to 0.22 μm syringe filter. D-/L-lactate assay kit (BioAssay Systems, USA) was used to quantify the amount of lactic acid secreted from *Lactobacillus*, following the manufacturer’s instruction. Briefly, CFS was loaded into 96-well flat bottom, transparent plate (SPL) and reacted with reaction mixture followed by color development detected at 565 nm (OD_565nm_). For the assay, the dilution factor was optimized so that the measurements remained within the linear range of the standard curve. 150-fold dilute and 30-fold dilute were used to quantify L-lactate and D-lactate respectively. The LDH reduced water-soluble tetrazolium to formazan (OD_565nm_). Lactate concentration was calculated based on the standard curve consisting of OD_565nm_ value and lactic acid concentration (mM). Total amount of lactate was calculated by summation of D-/L-lactic acid quantity.

### Lactate Dehydrogenase-Based Cell Cytotoxicity Assay

Caco-2 cell (ATCC HTB-37) was revived from cryo-stock and cultured in complete media composed of MEM (WELGENE, Republic of Korea) supplemented with 20% (v/v) Fetal Bovine Serum (SeraPrime, USA) and 1% (w/v) penicillin-streptomycin (WELGENE) at 37°C with 5% (v/v) CO_2_. Caco-2 cell was prepared as follows; Media were changed to antibiotic-free one two passages before cell seeding. At 90% confluency, cell was washed twice with DPBS (GenDEPOT) and detached by topical treatment of Trypsin-EDTA solution (WELGENE) at 37°C for 7 min. Trypsin was inactivated by adding equal volume of complete media. Cell containing medium was centrifuged at 4°C, 400 ×*g* for 5 min to harvest cells. Live cells were manually counted using hemocytometer (Labtech, Republic of Korea) after staining with Trypan blue (WELGENE) under light microscope. Caco-2 cells were inoculated on 96-well flat bottom, transparent plates (SPL) and grown at 80% confluency, approximately 5 × 10^4^ cells/well. Bacterial suspensions were added to each well to achieve at final concentrations of 10^7^, 10^8^, and 10^9^ CFU/well and incubated for 24 h. Secreted LDH from of Caco-2 cells were measured using EZ-LDH cell cytotoxicity assay kit (DoGenBio, Korea) based on the colorimetric value at OD_450nm_, which is proportional to LDH concentration following manufacturer’s instruction. *Escherichia coli* O157:H7 ATCC 43895 known to induce epithelial cell death [31] and LGG were tested as virulent and non-virulent control strains, respectively.

### Probiotic Stability Evaluation

**Oxygen tolerance in aerobic condition.** Probiotic candidates were inoculated at 1% (v/v) ratio to 150 μl of MRS-c broth in 96-well flat bottom, transparent plate (SPL). They were incubated either in anaerobic condition at static state or aerobic condition with orbital shaking. All groups were incubated at 37°C for 24 h without any interruption and OD_600nm_ was measured at 0 h and 24 h using Spark microplate reader (TECAN, Switzerland).

**Heat tolerance in various temperatures.** Probiotic candidates in MRS-c broth (10^9^ CFU/ml) were incubated at three different temperatures (37°C, 45°C, and 60°C) for 3 h under anaerobic conditions respectively. After heat treatment, samples were serially diluted at 10-fold ratio and inoculated on MRS-c agar.

**Survival in simulated gastric and bile acids.** Simulated Gastric acid (sGA) and simulated Bile acid (sBA) were prepared for the assay. sGA was composed of 0.073% (w/v) sodium chloride (Junsei, Japan), 0.0052% (w/v) potassium chloride (Daejung, Republic of Korea), 0.0378% (w/v) Sodium biocarbonate (Daejung), and 0.03% (w/v) pepsin (Sigma-Aldrich) dissolved in distilled water and the pH was adjusted to 2.5 using hydrogen chloride (Samchun, Republic of Korea). sBA was made of 0.01% (w/v) pancreatin (Daejung) and 0.03% (w/v) bile salts (Sigma-Aldrich) dissolved in distilled water, with pH adjusted to 8.0 using sodium hydroxide (Daejung). 1 × 10^9^ CFU/ml of test groups were incubated anaerobically in sGA at 37°C for 3 h. After that, cells were washed twice with DPBS (GenDEPOT) and sBA was added to simulate sequential exposure of probiotics in digestive tract followed by incubation at 37°C for 3 h. The equal amounts of control groups were exposed to DPBS (GenDEPOT) instead of sGA and sBA under the same culture conditions. After exposure, bacterial cells were washed with DPBS (GenDEPOT) and inoculated onto MRS-c agar.

**Adhesion ability to gut epithelial cell.** Caco-2 cell for adhesion assay was prepared as mentioned before in LDH assay with some modifications. For the adhesion assay, a density of 4 × 10^5^ cells/well in 12-well plates (SPL) was prepared. Probiotic candidates and control strain resuspended in complete cell culture media were loaded onto Caco-2 cell apically at the concentration of 10^8^ CFU/well and incubated for 2 h. After that, cell culture medium containing non-adherent bacteria was removed by washing method with DPBS (GenDEPOT) twice. Attached bacteria were harvested using 0.1% (v/v) Triton X-100 (Sigma-Aldrich) solution diluted with DPBS (GenDEPOT) at 37°C for 10 min. A mixture of cells and bacteria was diluted with DPBS (GenDEPOT) at 10-fold ratio and then inoculated on MRS-c agar.

### Probiotic Functional Analysis

**Bacterial lipase activity test.** Lipase activity was measured using Lipase assay kit (Abcam, England) following manufacturer’s protocol. 5 × 10^8^ CFU of bacterial cells were prepared by centrifugation at 11,400 ×*g* for 3 min followed by duplicate cell wash with DPBS (GenDEPOT). Assay buffer (300 μl) was added to bacterial cells and lysed by Bead Mill Homogenizer (Omni, USA) at 4.5 m/s for 30 sec for twice with 1 min on ice in between. After that, 50 μl of lysate supernatant was taken after centrifugation at 4°C, 11,400 ×*g* for 5 min, and loaded into 96-well flat bottom, transparent plate (SPL). Finally, the reaction mixture (100 μl) for the color development was added. Briefly, chromophore formed from a triglyceride substrate by bacterial lipase activity was detected in colorimetric assay at OD_570nm_. The OD_570nm_ was measured every 2 min using Spark microplate leader (TECAN). Given that enzymatic reactions exhibit high initial rates followed by a transition toward equilibrium, lipase activity was assessed at multiple time intervals. The linear increasement periods of each strain were predicted by linear regression method using a package ‘segmented’ [32] in R studio (ver.2025.09.2). The ΔOD_570nm_ was calculated by subtracting minimal OD_570nm_ from maximum OD_570nm_ in linear range and then converted to total quantity of lipid hydrolysis based on standard curve. After that, ΔOD_570nm_ was converted to lipase activity (mU/ml) in combination with total reaction time to represent the lipase activity.

**Bacterial proteolytic activity test.** A colorimetric assay using Ortho-phthalaaldehyde (OPA; Sigma-Aldrich) was used to evaluate proteolytic activity of probiotic candidates [33]. This method works by the reaction of OPA with free amino groups that are exposed when proteins are hydrolyzed by bacterial proteolytic activity. Modified MRS (mMRS) medium composed of 2% (w/v) dextrose (Samchun), 0.01% (w/v) proteose peptone no.3 (Difco), 0.5% (w/v) sodium acetate (Sigma-Aldrich), 0.2% (w/v) dipotassium phosphate (Sigma-Aldrich), 0.01% (w/v) magnesium sulfate (Junsei), 0.005% (w/v) manganese sulfate (Daejung), and 1% (v/v) Tween 80 (Samchun) was prepared to evaluate proteolytic activity. Four types of protein, milk protein concentrate (MPC), whey protein concentrate (WPC), casein, and soy protein, were added to mMRS at final concentration of 3% (w/v), respectively. Protein-containing culture media were sterilized by boiling at 80°C for 10 min. Probiotic candidates were inoculated at 1% (v/v) ratio and incubated at 37°C for 48 h. OPA reagent was made by dissolving 3.81% (w/v) disodium tetraborate decahydrate (Sigma-Aldrich), 0.1% (w/v) sodium dodecyl sulfate (Sigma-Aldrich), 0.08% (w/v) OPA (Sigma-Aldrich), and 0.088% (w/v) dithiothreitol (Sigma-Aldrich) to distilled water. 200 μl of each cell free supernatant and uninoculated mMRS was mixed with 600 μl of 110 mM trichloroacetic acid (Sigma-Aldrich) and centrifuged at 2,000 ×*g* for 30 min. After that, 10 μl of supernatant was added to 180 μl of OPA reagent in 96-well flat bottom, transparent plate (SPL). Plates were incubated at 37°C and OD_340nm_ at 30 min was measured. Proteolytic activity (%) was compared relative to that of proteinase K (100 μg/ml) as a standard.

**Lactose utilization ability test.** The composition of basal MRS medium (BMC) [34] was as follows; 1% (w/v) proteose peptone no.3 (Difco), 0.5% (w/v) yeast extract (Difco), 0.1% (w/v) potassium phosphate dibasic anhydrous (Daejung), 0.5% (w/v) sodium acetate trihydrate (Daejung), 0.2% (w/v) ammonium citrate dibasic (Daejung), 0.02% (w/v) magnesium sulfate heptahydrate, 0.0037% (w/v) manganese sulfate monohydrate, 0.1%(v/v) tween 80 (Samchun), 0.05% (w/v) L-cysteine hydrochloride (Sigma-Aldrich). The glucose (BMCG) and lactose (BMCL) medium were made by supplementation of 1% (w/v) Glucose (Samchun) or D-lactose (Sigma-Aldrich) to BMC with 0.22 μm filter sterilization, respectively. For the growth curve analysis, probiotic candidates were incubated in 150 μl of each BMC, BMCG, and BMCL broth in 96-well flat bottom, transparent plate (SPL), followed by OD_600nm_ measurement at every 3 h using SPARK microplate reader (TECAN). In lactose fermentation analysis, probiotic candidates were inoculated onto BMC, BMCG, and BMCL agar supplemented with 0.1% (w/v) bromocresol purple (Daejung)-BMCp, BMCGp, and BMCLp-by 0.22 μm filter sterilization. All inoculates were incubated until 48 h.

**Xylan utilization ability test.** Filtered sterile 5% (w/v) xylan (Tokyo Chemical Industry, Japan) [35] was added to BMC medium-BMCX. Probiotic candidates were incubated into 150 μl of BMC and BMCX in 96-well flat bottom, transparent plate (SPL), respectively. The OD_600nm_ was measured using Spark microplate reader (TECAN) at 0 h and 30 h. ΔOD_600nm (30 h – 0 h)_ was calculated in each medium, respectively and ΔΔOD_600nm_ was calculated by subtracting ΔOD_600nm_ of BMC from that of BMCX [36] as an indicator of xylan utilization.

## Results

### Isolation of *Lactobacillus* Strains from Fermented Food

*Lactobacillus* strains defined as probiotics by Korean MFDS were isolated from various food samples. Among them, three *Lactobacillus* strains were isolated from Ugok makgeolli, Nuruk yogurt, and Chorizo, respectively. They were identified to *L. plantarum* SK44-9, *L. fermentum* JL34-1, and *L. paracasei* JL32-5 using 16S rRNA sequencing analysis with BLASTN (data not shown).

### Safety Analysis

The safety of isolated strains was confirmed through determination of antibiotic resistance patterns, assessment of specific metabolic activities (D-lactate production, bile salt deconjugation and hemolytic activity), and cytotoxicity evaluation (lactate dehydrogenase (LDH) assay).

The MICs of three isolated strains against clinically relevant antibiotics are listed in Table 1. MIC values (mg/l) differed depending on the strain and the antibiotic tested; however, all remained at or below the EFSA cut-off limits. MIC value under cut-off indicates that bacterial strains are susceptible to the given antibiotics. Therefore, probiotic candidates have non-resistant phenotypes to antibiotics to fulfill the requirment of EFSA.

**Fig. 1:**
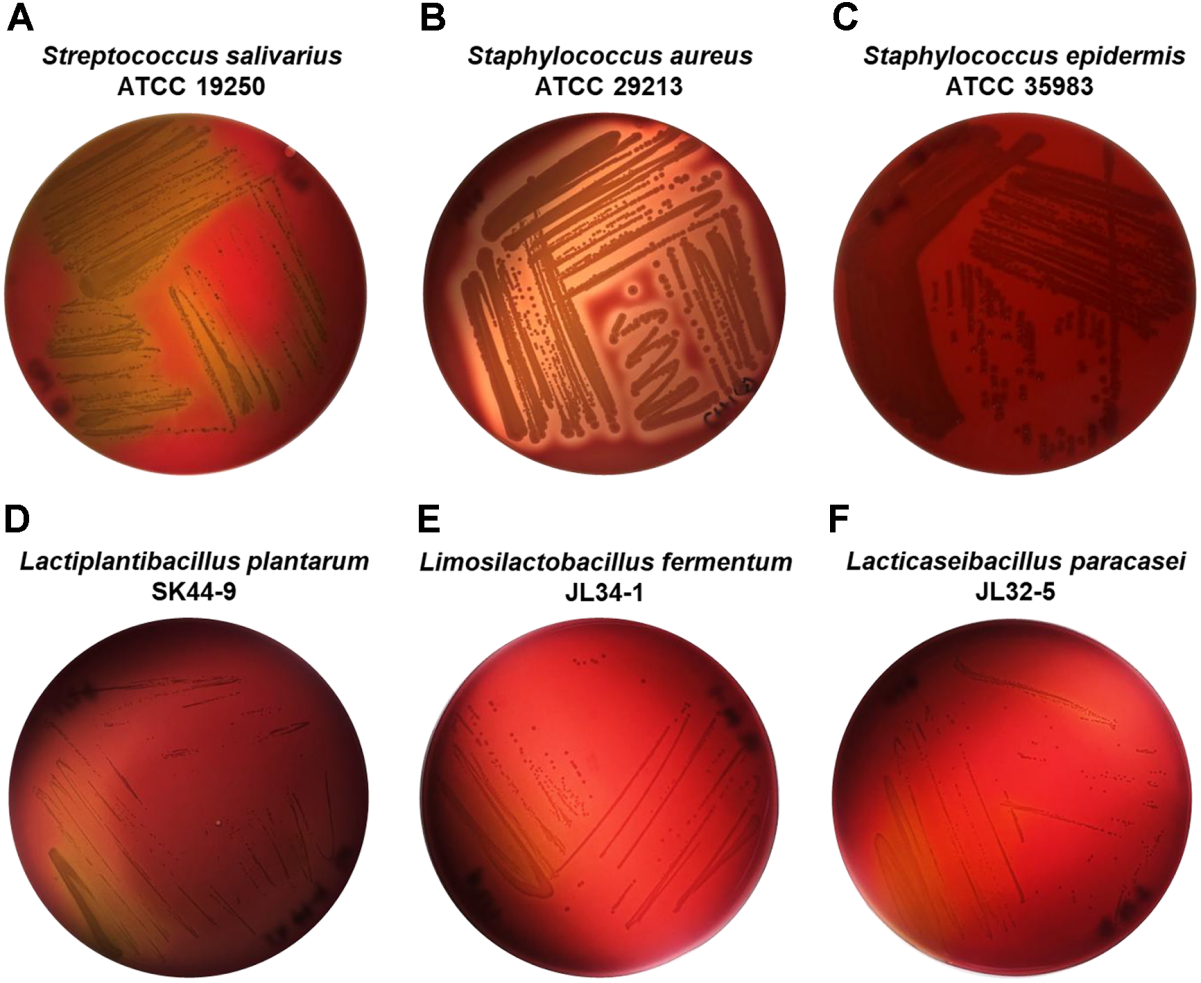
Colony morphologies of control strain and probiotic candidates after growth on Tryptic soy agar supplemented with 5% (v/v) sheep blood defibrinated for 36 h. (**A**) *Staph. salivarius* ATCC 19250. (**B**) *Strep. aureus* ATCC 29213. (**C**) *Staph. epidermis* ATCC 35983. (**D**) *L. plantarum* SK44-9. (**E**) *L. fermentum* JL34-1. (**F**) *L. paracasei* JL32-5.

In hemolysis test, three strains, *Strep. salivarius* ATCC 19250, *Staph. aureus* ATCC 29213, and non-hemolytic *Staph. epidermis* ATCC 35983, were selected and used as controls. ATCC 19250 (Fig. 1A) showed α-hemolysis characterized by greenish colonies, and ATCC 29213 (Fig. 1B) exhibited β-hemolysis indicated by clear zone surrounding colonies. However, ATCC 35983 (Fig. 1C) showed no hemolysis (γ-hemolysis). Likewise, SK44-9 (Fig. 1D), JL34-1 (Fig. 1E), and JL32-5 (Fig. 1F) showed neither greenish colonies nor clear zones, indicating that these strains showed γ-hemolysis. Therefore, these isolated three strains are safe from hemolysis activity.

**Fig. 2:**
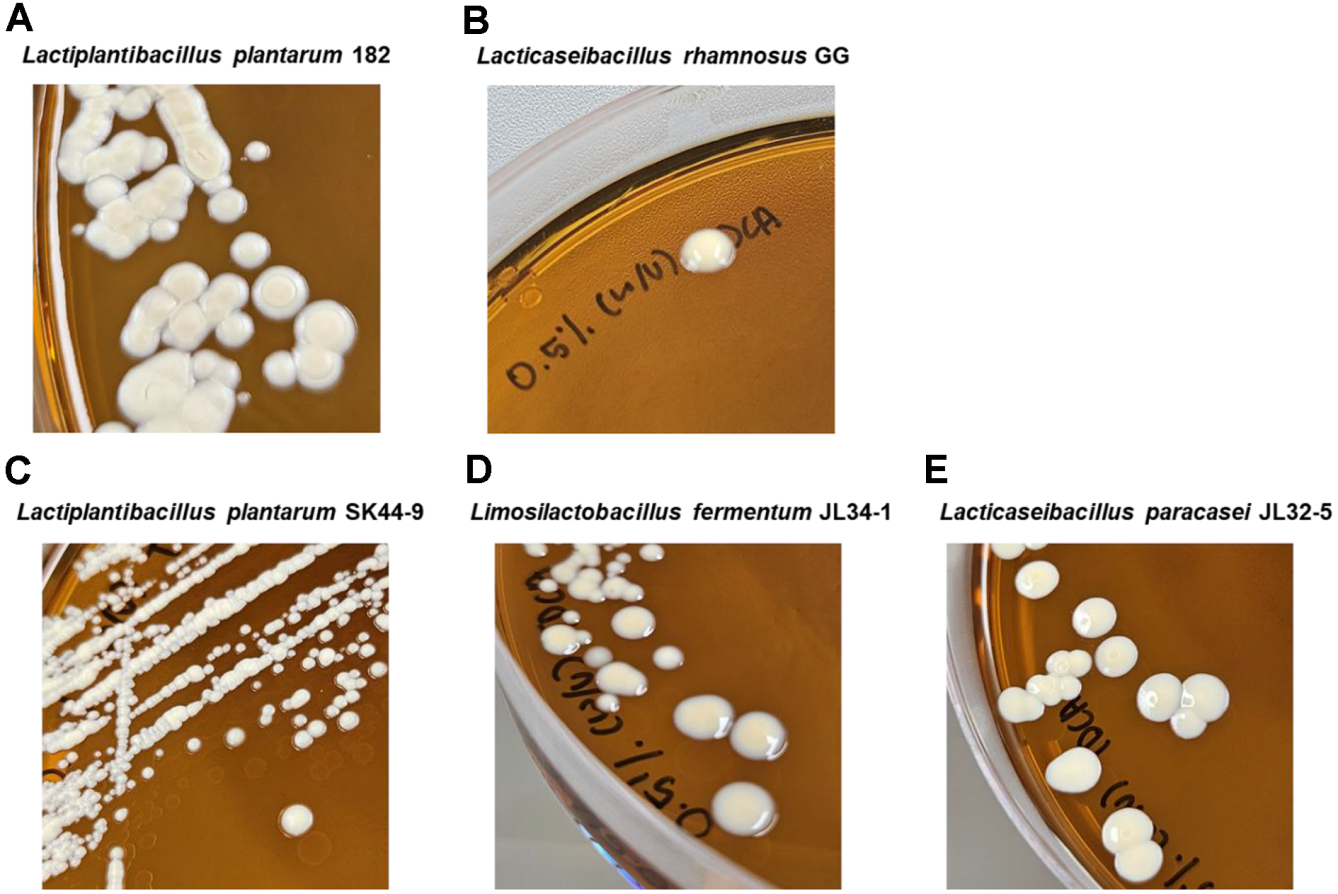
Representative colony morphologies of control strain and probiotic candidates on TDCA-included MRS-c (TDCA-MRS-c) agar. (**A**) *L. plantarum* 182 (**B**) LGG (**C**) *L. plantarum* SK44-9. (**D**) *L. fermentum* JL34-1. (**E**) *L. paracasei* JL32-5.

In BSH activity test, opaque colony with brown halo was regarded as active in bile salt deconjugation by enzymes secreted from bacteria. The control strain, *L. plantarum* 182, showed opaque, dry white colonies (Fig. 2A). In contrast, LGG (Fig. 2B) and three probiotic candidates (Fig. 2C-2E) showed translucent colonies. The distinct colony morphologies indicated that these isolated probiotic strains (SK44-9, JL34-1, and JL32-5) did not exhibit phenotypic BSH activity.

**Fig. 3:**
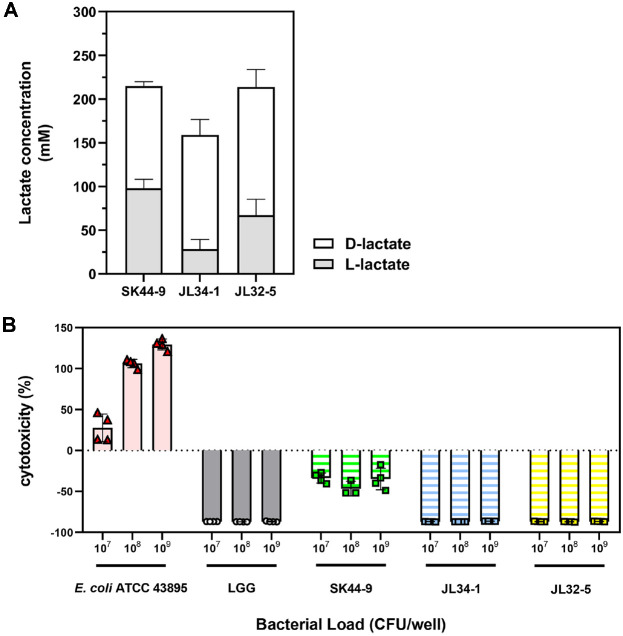
Lactate profile and cell cytotoxicity of probiotic candidates. (**A**) The amount of secreted D-lactic acid (white bar), L-lactic acid (light grey bar), and total lactic acid (stacked bar) in CFS is expressed in lactate concentration (mM). Data are presented as mean ± SEM (n = 3). (**B**) The relative cytotoxicity (%) based on released LDH from Caco-2 after exposure to control strain and probiotic candidates of various concentration (CFU/well). Complete cell lysis by cell lysis solution in LDH assay kit is expressed as 100% of cytotoxicity and normally released level of LDH was presented as 0% of cytotoxicity. Data are presented as mean ± SEM (n = 4).

Bacterial lactate was oxidized by NADH-producing lactate dehydrogenase which reduces colorless 3-(4,5-Dimethylthiazol-2-yl)-2,5-Diphenyltetrazolium Bromide (MTT) to violet-colored formazan (OD_565nm_). The amount of lactate was determined by OD_565nm_ value. The isolated strains (SK44-9, JL34-1, and JL32-5) showed ability to produce both L- and D-lactate (Fig. 3A), but the ratio of D- and L-lactate was different among the strains. SK44-9 produced 117 ± 5 mM L-lactate and 98 ± 10 mM D-lactate (L:D = 54:46), JL34-1 produced 130 ± 18 mM L-lactate and 29 ± 11 mM D-lactate (82:18), and JL32-5 produced 147 ± 20 mM L-lactate and 67 ± 18 mM D-lactate (70:30). These isolated strains produced more D-lactate than L-lactate as a major metabolite. However, the amount of total lactate was similar between SK44-9 (215 ± 14 mM) and JL32-5 (214 ± 22 mM), but higher than that of JL34-1 (159 ± 28 mM).

In cytotoxicity assay, over 100% of cytotoxicity indicates complete cell death induced by cell lysis solution. In contrast, a cytotoxicity value of 0% represents the baseline LDH release from live cells in the untreated control group. *E. coli* O157:H7, virulent as enterohemorrhagic *E. coli* (EHEC) against gut epithelium, exhibited cytotoxicity in dose-dependent manner. However, three isolated strains (SK44-9, JL34-1, and JL32-5) showed no cytotoxicity to Caco-2 cells at variable concentrations (Fig. 3B). Probiotic candidates and LGG induced lower LDH release than that of untreated cell. Therefore, all three strains exhibited no cytotoxicity on Caco-2 cells. Interestingly, LGG, JL34-1, and JL32-5 showed similar trends unlike SK44-9.

From these four safety evaluation tests, these three probiotic candidates were validated to possess safety profiles. Therefore, these strains were further evaluated of their stability in various stress conditions regarding commercial processing and human gut intestinal tract to investigate strain-specific stability properties in each stress condition.

### Stability Analysis

Thermotolerance and aerotolerance were determined under temperatures exceeding human body temperature and aerobic conditions, respectively. The potential survival of the probiotic candidates in the human gastrointestinal tract was evaluated *in vitro* through sequential exposure to gastric acid and bile salts, followed by assessment of their adhesion ability to intestinal epithelial cells

**Fig. 4:**
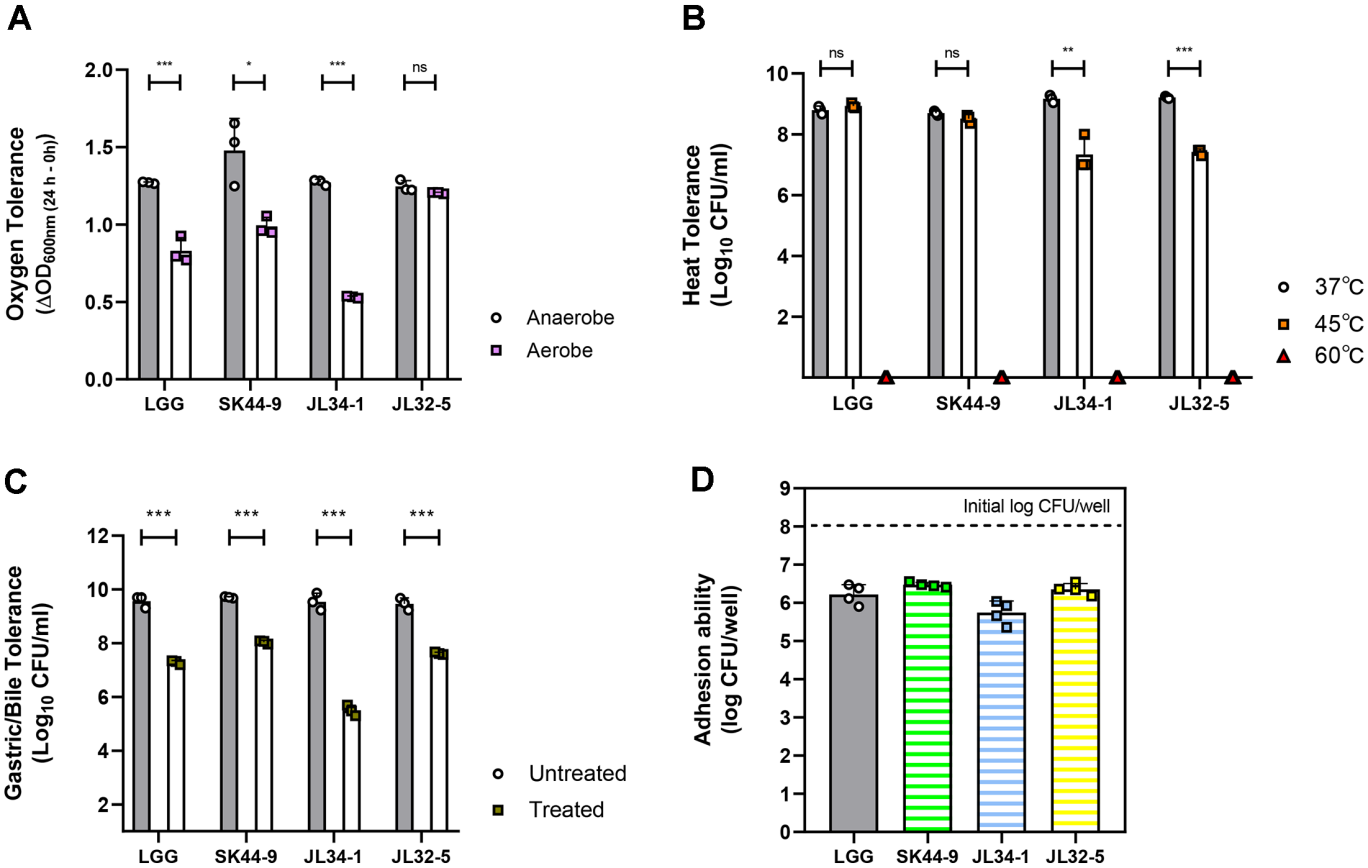
Relative survival at stress conditions and adhesion ability to gut epithelial cells. The survival rates at normal condition—37°C, anaerobic, and untreated (dark grey bar)–are comparable at each other. (**A**) Aerotolerance in aerobic culture condition (light purple points) presented as mean ± SEM (n = 3). (**B**) Thermotolerance at 45°C (orange points) and 60°C (red points) presented as mean ± SEM (n = 3). (**C**) Tolerance to sequential exposure of sGA and sBA (dark green points) presented as mean ± SEM (n = 3). (**D**) Adhesion ability (CFU/well) to Caco-2 presented as mean ± SEM (n = 4). Statistical significance was determined by multiple *t*-tests corrected by the Holm-sidak method. Asterisks represent as follows; *p < 0.05, **p < 0.01, ***p < 0.001 compared to normal condition group. ns denotes no significant difference; n.d. indicates values below the detection limit.

In aerotolerance test (Fig. 4A), probiotic candidates exhibited comparable ΔOD_600nm (24 h – 0 h)_ value in anaerobic culture. The ΔOD_600nm (24 h – 0 h)_ of aerobic culture was positive in probiotic candidates but lower than that of anaerobic culture. However, the growth of JL32-5 did not show a statistically significant difference in between aerobic and anaerobic conditions, indicating the higher aerotolerance than other probiotic candidates.

In thermotolerance test (Fig. 4B), LGG and probiotic candidates grew to around 9 log CFU/ml at 37°C-LGG, 8.8 ± 0.13 log CFU/ml; SK44-9, 8.7 ± 0.07 log CFU/ml; JL34-1, 9.2 ± 0.12 log CFU/ml; JL32-5 9.2 ± 0.04 log CFU/ml. At 45°C, the survival of LGG (8.9 ± 0.08 log CFU/ml) and SK44-9 (8.5 ± 0.14 log CFU/ml) was comparable with that of 37°C. However, JL34-1 (7.3 ± 0.58 log CFU/ml) and JL32-5 (7.4 ± 0.1 log CFU/ml) showed significant decreases at 45°C. The survival at 60°C was not detected from all tested strains. These findings suggest that probiotic candidates showed thermotolerance, but SK44-9 showed the highest thermotolerance, compared to JL34-1 and JL32-5.

The amount of live bacterial cells after sequential exposure to sGA and sBA exhibited the gastrointestinal tolerance (Fig. 4C). The survival of untreated groups was comparable with each other—LGG, 9.6 ± 0.23 log CFU/ml; SK44-9, 9.7 ± 0.03 log CFU/ml; JL34-1, 9.5 ± 0.32 log CFU/ml; JL32-5 9.5 ± 0.22 log CFU/ml. However, their survival in simulated gastrointestinal environment was different and lower than that of untreated groups—LGG, 7.3 ± 0.07 log CFU/ml; SK44-9, 8 ± 0.07 log CFU/ml; JL34-1, 5.5 ± 0.2 log CFU/ml; JL32-5, 7.6 ± 0.05 log CFU/ml. Compared to LGG, SK44-9 and JL32-5 showed higher tolerance to gastrointestinal environment

Adherence of bacterial cells (CFU/well) to Caco-2 cells indicates colonization potential of the isolated strains to gut epithelium (Fig. 4D). LGG and probiotic candidates demonstrated similar intestinal adhesion rates—LGG, 6.2 ± 0.26 log CFU/ml; SK44-9, 6.5 ± 0.06 log CFU/ml; JL34-1, 5.7 ± 0.3 log CFU/ml; JL32-5, 6.4 ± 0.15 log CFU/ml. Compared to LGG, both SK44-9 and JL32-5 are expected to have an adhesion potential at least equivalent to that of LGG.

### Lipase Activities of Isolated Strains Showed Strain-Specificity

**Fig. 5:**
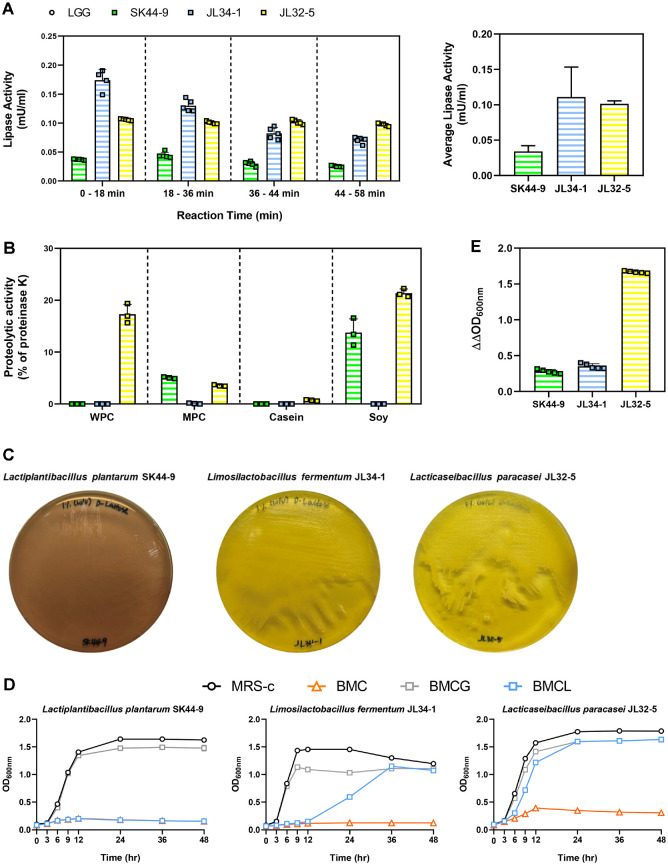
Digestive supporting function of isolated strain. (**A**) Bacterial lipase activity (mU/ml) across four reaction periods and the average activity over the entire reaction. Data are presented as mean ± SEM (n = 5 and n = 20, respectively). (**B**) Proteolytic activities quantified as a percentage relative to the activity of proteinase K (% of proteinase K) against four types of protein substrates—milk protein concentrate (MPC), whey protein concentrate (WPC), casein, and soy. Data are presented as mean ± SEM (n = 3) (**C**) Lactose fermentation ability in BMCL-p agar. (**D**) Bacterial growth curve dependent on time and culture media. Data are presented as mean ± SEM (n = 3). MRS-c (black line), MRS supplemented with 0.05% (w/v) L-cysteine hydrochloride; BMC (orange line), basal MRS-c; BMCG (light grey line), basal MRS-c supplemented with 1% (w/v) Glucose; BMCL (light blue line), basal MRS-c supplemented with 1% (w/v) lactose.

The phases during which the probiotic candidate’s lipase activity increased linearly are presented in Table S1. Considering the overlapped regions among the four strain-specific linear segments, the bacterial lipase activities were evaluated within four unified intervals: 0–18 min (Q1), 18–36 min (Q2), 36–44 min (Q3), and 44–58 min (Q4) (Fig. 5A). Among the tested strains, JL34-1 exhibited similar pattern with the highest lipase activity observed in Q1 followed by a gradual decrease as the reaction progressed. In specific, JL34-1 degraded triglyceride at rates of 0.174 ± 0.018 mU/ml in Q1, 0.130 ± 0.01 mU/ml in Q2, 0.082 ± 0.01 mU/ml in Q3, and decreased up to 0.07 ± 0.005 mU/ml in Q4. In contrast, JL32-5 and SK44-9 maintained relatively constant enzyme activity across all intervals. JL32-5 exhibited 0.106 ± 0.001, 0.101 ± 0.002, 0.102 ± 0.004, and 0.097 ± 0.002 mU/ml from Q1 to Q4, while SK44-9 showed 0.037 ± 0.001, 0.044 ± 0.005, 0.030 ± 0.004, and 0.025 ± 0.001 mU/ml from Q1 to Q4, respectively. On comparison of the mean values, JL34-1 and JL32-5 exhibited comparable levels of activity, 0.11 ± 0.04 and 0.1 ± 0.004 mU/ml, respectively, which were higher than that of SK44-9 (0.034 ± 0.008 mU/ml, mean value). High lipase activity reflects an enhanced capacity of lipid hydrolysis. Therefore, JL34-1 and JL32-5 may possess the potential for lipid-degrading functionality, suggesting that lipase activity may be strain-specific.

### JL32-5 Exhibited the Broad Proteolytic Activity

Bacterial proteolytic activity against variable protein types were expressed as an average percentage relative to that of proteinase K (Fig. 5B). Probiotic candidates carrying proteolytic activity exhibited relatively low level than that of proteinase K (100%). SK44-9 showed proteolytic activity only against milk protein concentrate (MPC; 5 ± 0.2%) and soy protein (13.8 ± 2.6%), but no proteolytic activity against whey protein concentrate (WPC) and casein. JL34-1 showed no proteolytic activity against all protein sources. However, JL32-5 showed proteolytic activities against MPC (3.5 ± 0.2%), soy protein (21.4 ± 0.8%), WPC (17.3 ± 1.9%), and casein (0.7 ± 0.1%,). Therefore, among three tested strains, JL32-5 exhibited the highest proteolytic activity, suggesting the potential protein-degrading probiotic strain.

### JL32-5 Showed the Highest Lactose Utilization Capacity

The appearance of a yellow color of BMCLp was interpreted as positive result of lactose utilization. After 48 h incubation, the color of SK44-9-inoculated plate remained unchanged. Meanwhile, JL34-1 and JL32-5 showed color development from purple to yellow (Fig. 5C). Therefore, the lactose utilization activities of JL34-1 and JL32-5 were confirmed in phenotypic aspects. In fact, the color change of JL32-5 (24 h) was faster than that of JL34-1 (48 h) indicating higher lactose utilization ability of JL32-5 than that of JL34-1.

To further understand the relative lactose degradation ability, the growth of probiotic candidates in BMC medium supplemented with lactose as a sole carbon source (BMCL) were investigated. As a result, these probiotic candidates grew well in MRS-c and BMCG media, but not in BMC medium. While JL32-5 and JL 34-1 grew in BMCL medium, SK44-9 exhibited no growth in the same medium. Interestingly, the growth of JL32-5 was faster than that of JL34-1 (Fig. 5D), supported by required time to reach related stationary phase (JL32-5, 12 to 24 h; JL34-1, over 24 h). These results support that JL32-5 has the highest lactose utilization ability.

### JL32-5 Demonstrated the Highest Xylan Utilization Activity

Xylan utilizing ability is proportional to ΔΔOD_600nm_, an experimental marker of bacterial growth using xylan as a sole carbon source. Noting this, all test strains exhibited xylan utilizing capacity (Fig. 5E). Among all tested strains, JL32-5 showed the notable growth compared to other probiotic candidates.

## Discussion

This study provides phenotypic evidence that *L. paracasei* JL32-5 is the potential digestion-supporting probiotic candidate. Through systematic screening of isolates, JL32-5 was identified as the best strain exhibiting the most balanced and superior digestive functions across essential dietary macronutrients-including lipids, proteins, lactose, and xylan. The ability to degrade triglyceride, a major form of dietary lipid [37], was interpreted as a surrogate marker of general lipid hydrolysis. Proteolytic activity was confirmed against both mammalian protein (WPC, MPC, casein), and vegetarian protein (soy). To target lactose intolerance, a representative maldigestion disorder, lactose utilization was evaluated and confirmed as another key digestive function. In addition, xylan-a non-digestible carbohydrate and a structural component of the plant cell wall-was used to assess bacterial utilization of a dietary fiber. Because both lactose and xylan are composed of monosaccharide, the growth of JL32-5 on lactose or xylan was considered indirect evidence of substrate degradation, with the expectation that released monosaccharides such as glucose would support bacterial growth. Through these digestive functions, vulnerable individuals with lactose intolerance or general maldigestion may benefit from the administration of digestion-supporting probiotics to alleviate symptoms such as bloating and diarrhea. Considering functional potential across isolates, JL32-5 exhibited a well-balanced digestive profile, making it more suitable for comprehensive probiotic functionality than SK44-9 and JL34-1 possessing narrow activities [38]. of SK44-9 and JL34-1. Moreover, JL32-5 demonstrated the highest enzymatic activity across all tested substrates, consistent with the known capabilities of lactobacilli from fermented foods to produce digestive enzymes with strain-specific beneficial effects [39].

Regarding general probiotic characteristics, JL32-5 also fulfilled key criteria, including stress tolerance, gastrointestinal survival, adhesion capacity, and safety. JL32-5 exhibited the highest aerotolerance among the tested strains. Although SK44-9 exhibited greater thermotolerance, gastric resistance, and adhesion, the absolute values for JL32-5 were comparable and not significantly different. In addition, the negative cytotoxicity observed in Caco-2 cells treated with JL32-5 suggests a potential protective effect, as treated cells displayed lower cell death than untreated controls. This result might be interpreted as a no cytotoxic of isolated strains to cells. However, these three isolated probiotic candidates including JL32-5 produced D-lactic acid as a major metabolite. While people primarily metabolize L-isomer form with lactate [40], accumulation of D-lactate in the colon may occur in susceptible individuals. Though D-lactic acidosis is a rare metabolic complication in humans, excessive accumulation can reduce intestinal pH and contribute to clinical symptoms. Therefore, excessive administration of JL32-5 should be carefully considered in individuals at risk of D-lactate–associated complications.

While this study suggests JL32-5 as a potential probiotic source to alleviate maldigestions, several limitations should be acknowledged. The systematic selection employed in this study, while well-established, is relied on phenotypic assays. Therefore, the molecular mechanism underlying substrate degradation remains to be elucidated. Two major hypotheses warrant investigation. (1) The macronutrients are uptaken by isolated bacteria and metabolized by intracellular enzymes, or (2) secreted bacterial enzymes degrade macronutrients extracellularly. These hypothesis may be addressed through metabolomic analysis of cell lysates to identify intracellular metabolites derived from macronutrient utilization and enzymatic assays using the cell-free supernatant to determine extracellular enzyme activity. Such analyses could also help identify additional bioactive compounds, including short-chain fatty acids (SCFAs) or xylo-oligosaccharides [41]. In addition, genomic analysis based on complete genome sequence of JL32-5 should be applied to characterize the genetic basis of its digestive functions and to identify putative enzymes. In respect of the probiotic safety, cross validation of phenotypic results through genomic assay is a critical prerequisite for probiotic application. Due to the potential presence of mobile virulence factors that can be transferred to other gut microbiota through horizontal gene transfer [42], confirming the absence of virulence factors will further strengthen the safety profile of JL32-5. With subsequent molecular validation and *in vivo* evaluations, JL32-5 may be further developed as a commercial probiotic strain, capable of preventing or alleviating gastrointestinal symptoms such as flatulence, increased intestinal wall tension, and bloating [43], thereby contributing to improved digestive health.

This study demonstrates that *L. paracasei* JL32-5 exerts a comprehensive digestive functionality across various macronutriets. The strain exhibited a broad enzymatic repertoire, including triglyceride hydrolysis, proteolysis against both animal- and plant-derived proteins, lactose utilization, and xylan degradation. Such multifaceted digestive mechanisms indicates a robust enzymatic system capable of supporting nutrient breakdown in the gastrointestinal tract. By facilitating more efficient hydrolysis of dietary components, JL32-5 may reduce the accumulation of unmetabolized nutrients that are often fermented by resident gut microbes, thereby preventing subsequent production of gas and organic acids responsible for gastrointestinl discomfort. These findings lay a strong conceptual and experimental foundation for the future clinical advancement of JL32-5 as a novel, multifunctional probiotic strategy for maintanence of overall gastrointestinal health.

## Figures and Tables

**Table 1:** Minimum Inhibitory Concentrations (MICs) results of probiotic candidates.